# A simple test of “Kami-Tsumami” (Paper Tug) for pinch strength screening among community-dwelling older adults

**DOI:** 10.1016/j.tjfa.2025.100060

**Published:** 2025-07-01

**Authors:** Weida Lyu, Tomoki Tanaka, Katsuya Iijima

**Affiliations:** a Institute of Gerontology, The University of Tokyo, Tokyo, Japan; b Institute for Future Initiatives, The University of Tokyo, Tokyo, Japan

Dear Editor

Pinch strength, which represents the force generated between the thumb and fingers, is an essential measure of hand function and is closely linked to overall muscle and grip strength in older adults [1]. In particular, reduced pinch strength is linked to difficulty performing everyday tasks, indicating its critical role in maintaining independence and quality of life among older adults [2]. Therefore, a decline in pinch strength may suggest early functional impairment. In aging societies like Japan, where many older adults live independently in communities, early detection of physical decline is crucial for timely intervention. However, existing assessments of pinch strength typically require specialized equipment and trained personnel, limiting their feasibility in community settings. To address these barriers, a simple, practical, and accessible method for evaluating pinch strength is needed—particularly one that can be used in everyday environments and by non-professionals.

To address this, we developed the “Paper Tug” (Kami-Tsumami in Japanese) test, an innovative method utilizing a standard sheet of postcard to assess pinch strength. As the Supplementary Fig. 1 shows, in the Kami-Tsumami test, the participant and the tester were seated, facing each other, each holding one end of a standard postcard (B6: 125 mm, 176 mm) using their dominant hand's thumb and index finger pads. The participant’s upper arm was kept close to the torso, with the forearm positioned horizontally in front of the body. The tester was a healthy 30-year-old man (height: 185 cm; weight: 80 kg). Upon the verbal cue “start,” the participant was instructed to attempt to pull the postcard out of the tester’s grip within three seconds. The outcome was classified as “capable” if the participant successfully pulled the paper away or “incapable” otherwise. This study aims to explore the association between Kami-Tsumami test outcomes with actual pinch strength, and other physical function indicators among community-dwelling older adults aged 80 years and older.

This study included 343 community-dwelling older adults aged ≥80 from the seventh wave of the Kashiwa Cohort Study conducted in Kashiwa City, Chiba Prefecture, Japan [3]. A hydraulic pinch gauge measured the pinch strength between the thumb and index finger pads (Sakai Medical Co., Ltd., Japan). Sarcopenia was assessed following the 2019 Asian Working Group criteria (AWGS2019) [4]. The status of age, sex, body mass index (BMI), educational level, Mini-Mental State Examination (MMSE) scores, depression status (GDS15), physical function, and chronic diseases are summarized in Supplementary Table S1. Analyses were conducted using IBM SPSS software (version 29.0), with significance defined as *p* < 0.05. Ethical approval was granted by the Ethics Committee of the University of Tokyo (approval #23–304).

Of the 343 participants, 115 (33.4 %) completed the Kami-Tsumami test and were categorized as capable. They were significantly older, but no differences in chronic disease status were observed. Sex-stratified analyses revealed that Kami-Tsumami performance was significantly associated with pinch strength in both men and women. Among men, capable participants showed higher BMI, ASMI, grip strength, and pinch strength, while among women, they exhibited higher BMI, grip strength, pinch strength, and gait speed. No significant associations were found with MMSE or GDS15 (Supplementary Table S1).

Based on the results of multiple regression analyses, pinch strength was significantly associated with Kami-Tsumami test in both men and women. However, grip strength was significantly associated with only in women, while ASMI showed a significant association exclusively in men. In contrast, no significant correlations were observed between the Kami-Tsumami test and gait speed, time up and go test, or the sit-to-stand test (Table 1). Moreover, to evaluate the discriminatory ability of the Kami-Tsumami test in identifying low pinch strength, we conducted a Receiver Operating Characteristic (ROC) analysis using the age- and sex-specific the lowest 20th percentile as the cutoff, following established methods [1]. The area under the curve (AUC) was 0.702 (95 % CI: 0.617–0.787, *p* < 0.001), indicating acceptable discrimination (Figure S2). These findings support the practical utility of the Kami-Tsumami test as a low-cost, binary screening tool to detect reduced pinch strength in older adults. Additionally, the Kami-Tsumami test did not demonstrate significant associations with sarcopenia or its three major components according to the binomial logistic regression analysis (Supplementary Table S2).

**Table 1:** Associations between the Paper Tug Test (Kami-Tsumami test), pinch strength, and physical function.

Outcome	mean ± SD	Univariate analysis			Multivariate analysis		
		β	95 %CI for β	*P*	β	95 %CI for β	*P*
**Women (*n* = 204)**							
Pinch Strength, kg	6.5 (±1.8)	1.28	(0.70–1.85)	***<0.001***	1.26	(0.64–1.88)	***<0.001***
Grip Strength, kg	19.8 (±3.9)	1.82	(0.55–3.10)	***<0.001***	1.99	(0.76–3.23)	***0.002***
Gait Speed, m/s	1.3 (±0.3)	0.09	(0.00–0.17)	***0.420***	0.08	(0.00–0.16)	***0.058***
Timed Up and Go test, s	6.5 (±1.9)	−0.51	(−1.15–0.12)	***0.113***	−0.51	(−1.13–0.12)	***0.111***
ASMI, kg/m^2^	5.8 (±0.6)	0.05	(−0.17–0.27)	***0.644***	−0.11	(−0.27–0.50)	***0.175***
**Men (*n* = 139)**							
Pinch Strength, kg	8.5 (±2.1)	2.14	(1.52–2.75)	***<0.001***	1.78	(1.11–2.44)	***<0.001***
Grip Strength, kg	30.4 (±5.2)	2.91	(1.27–4.56)	***<0.001***	0.59	(−1.00–2.19)	***0.735***
Gait Speed, m/s	1.4 (±0.2)	0.03	(−0.05–0.10)	***0.470***	0.02	(−0.60–0.10)	***0.610***
Timed Up and Go test, s	5.8 (±1.2)	−0.52	(−0.91–0.13)	***0.010***	−0.28	(−0.70–0.14)	***0.191***
ASMI, kg/m^2^	7.2 (±0.7)	0.42	(0.20–0.63)	***<0.001***	0.19	(0.01–0.38)	***0.043***

Abbreviations*:* BMI, body mass index; ASMI, appendicular skeletal muscle mass index.

Data are shown as means (±standard deviations).

Multivariate analysis Adjusted model included as covariates: age, sex, body mass index, ASMI, living arrangements, education duration, living arrangements, education duration, mini-mental state examination, and Geriatric Depression Scale-15.

The Kami-Tsumami test offers unique advantages for community screening: it is simple, equipment-free, and easy to interpret, making it suitable for home visits and local health events. Compared to tools like the Yubi-wakka [3] or Newspaper Tear-Off tests [5], which assess general muscle mass or weakness, this test focuses on fine motor control and pinch strength—critical for daily independence. Notably, two-thirds of participants could not complete the test, indicating its potential to identify high-risk individuals who might otherwise be overlooked. For these individuals, the test may prompt timely referral to rehabilitation or strength-maintenance programs. Despite its practicality, the current version of the test has some limitations, including variability in the tester’s grip force and the binary outcome which may oversimplify the spectrum of hand strength. Moreover, longitudinal studies are needed to verify its predictive validity for frailty-related outcomes and to assess its applicability across different cultural and community contexts.

## Funding

This work was supported by JSPS KAKENHI (grant no.:24K23744); JSPS KANKENHI (24KK0169); a Health and Labor Sciences Research Grant (grant no.: H24-Choju-Ippan-002) provided by the Ministry of Health, Labor, and Welfare of Japan; the Japan Agency for Medical Research and Development (grant no.: JP21km0908001); the Institute for Health Economics and Policy, Japan.

## Disclosure statement

On behalf of all authors, the corresponding author states that there is no conflict of interest.

## CRediT authorship contribution statement

**Weida Lyu:** Writing – original draft, Validation, Software, Resources, Methodology, Investigation, Funding acquisition, Data curation, Conceptualization. **Tomoki Tanaka:** Writing – review & editing, Supervision, Conceptualization. **Katsuya Iijima:** Writing – review & editing, Supervision, Project administration, Investigation, Funding acquisition.

## Appendix Supplementary materials

Image, application 1
