# Supplementary material for: A simple test of “Kami-Tsumami” (Paper Tug) for pinch strength screening among community-dwelling older adults
Source: J Frailty Aging. 2025 Jul 1;14(4):100060. doi: 10.1016/j.tjfa.2025.100060 (PMC12399246; doi:10.1016/j.tjfa.2025.100060)
Supplement: Supplementary file 1 [file mmc1.docx]

**Figure S1. “Kami-Tsumami” (Paper Tug) Test.**

**Figure S2. Receiver operating characteristic curves to estimate the sensitivity and specificity of the Kami-Tsumami test for low pinch strength screening.**

| **Table S1.** Participant characteristics according to the Paper Tug Test (Kami-Tsumami test). | | | | |  |
| --- | --- | --- | --- | --- | --- |
|  | **Overall** | **Paper Tug Test (Kami-Tsumami test)** | | ***P ^*^*** |  |
|  |  | **Capable** | **Incapable** |  |  |
| No. of participants, n | 343 | 115 (33.4%) | 229 (66.6%) | ***-*** |  |
| ***Basic characteristics*** |  |  |  |  |  |
| Age, years | 83.1 (±5.8) | 83.6 (±7.0) | 82.9 (±5.1) | ***0.005^a^*** |  |
| Sex, women, n (%) | 204 (59.5%) | 65 (57.0%) | 75 (32.8%) | ***<0.001^b^*** |  |
| Education year, years | 13.2 (±2.6) | 13.0 (±2.6) | 13.3 (±2.7) | ***0.360^a^*** |  |
| Living alone, n (%) | 62 (18.1%) | 20 (17.4%) | 42 (18.4%) | ***0.815^b^*** |  |
| MMSE, score | 29 [27-30] | 28 [27-30] | 29 [27-30] | ***0.072^c^*** |  |
| GDS15, score | 3 [1-5] | 3 [1-7] | 2 [1-5] | ***0.078^c^*** |  |
| ***Physical function*** |  |  |  |  |  |
| Body mass index, kg/m^2^ | 22.6 (±2.8) | 21.7 (±2.8) | 23.0 (±2.6) | ***<0.001^a^*** |  |
| Men | 22.9 (±2.6) | 22.1 (±2.5) | 23.2 (±2.6) | ***0.016^a^*** |  |
| Women | 22.1 (±2.9) | 21.5 (±3.0) | 22.6 (±2.8) | ***0.026^a^*** |  |
| Appendicular SMI, kg/m^2^ | 6.6 (±1.0) | 6.2 (±0.9) | 6.8 (±1.0) | ***<0.001^a^*** |  |
| Men | 7.2 (±0.7) | 6.9 (±0.7) | 7.3 (±0.7) | ***<0.001^a^*** |  |
| Women | 5.8 (±0.6) | 5.7 (±0.6) | 5.8 (±0.6) | ***0.562^a^*** |  |
| Grip strength, kg | 26.1 (±7.1) | 22.9 (±6.1) | 27.7 (±7.0) | ***<0.001^a^*** |  |
| Men | 30.4 (±5.2) | 28.2 (±4.3) | 31.1 (±5.3) | ***<0.001^a^*** |  |
| Women | 19.8 (±3.9) | 18.7 (±3.6) | 20.6 (±4.0) | ***0.015^a^*** |  |
| Pinch strength, kg | 7.7 (±2.2) | 6.3 (±1.4) | 8.4 (±2.2) | ***<0.001^*^*** |  |
| Men | 8.5 (±2.1) | 9.9 (±2.0) | 5.9 (±1.4) | ***<0.001^a^*** |  |
| Women | 6.5 (±1.8) | 9.0 (±2.1) | 7.1 (±1.9) | ***<0.001^a^*** |  |
| Gait speed, m/s | 1.3 (±0.2) | 1.3 (±0.3) | 1.4 (±0.2) | ***0.044^a^*** |  |
| Men | 1.4 (±0.2) | 1.3 (±0.3) | 1.4 (±0.2) | ***0.610^a^*** |  |
| Women | 1.3 (±0.3) | 1.3 (±0.3) | 1.4 (±0.2) | ***0.037^a^*** |  |
| Timed Up and Go test, s | 6.1 (±1.6) | 6.5 (±1.7) | 5.9 (±1.4) | ***<0.001^a^*** |  |
| Men | 5.8 (±1.2) | 6.2 (±1.6) | 5.7 (±1.1) | ***0.011^a^*** |  |
| Women | 6.5 (±1.9) | 6.8 (±1.8) | 6.3 (±1.9) | ***0.021^a^*** |  |
| ***Comorbid conditions, n (%)*** |  |  |  |  |  |
| Hypertension | 173 (50.3%) | 63 (54.8%) | 110 (48.0%) | ***0.238^b^*** |  |
| Diabetes mellitus | 42 (12.2%) | 10 (8.7%) | 32 (14.0%) | ***0.158^b^*** |  |
| Osteoporosis | 33 (9.6%) | 15 (13.0%) | 18 (7.9%) | ***0.124^b^*** |  |
| Dyslipidemia | 98 (28.5%) | 30 (26.1%) | 68 (29.7%) | ***0.484^b^*** |  |
| Malignant neoplasm | 60 (17.4%) | 22 (19.1%) | 38 (16.6%) | ***0.559^b^*** |  |
| Heart disease | 49 (14.2%) | 11 (9.6%) | 38 (16.6%) | ***0.078^b^*** |  |
| Stroke | 14 (4.1%) | 7 (6.1%) | 7 (3.1%) | ***0.180^b^*** |  |
| Chronic renal failure | 5 (1.5%) | 3 (2.6%) | 2 (0.9%) | ***0.205^b^*** |  |
| Abbreviations: MMSE, Mini-Mental State Examination; GDS15, Geriatric Depression Scale-15.  Data are shown as means (±standard deviations)  ^*^The differences in participant characteristics based on the “Paper Tug Test (Kami-Tsumami test)” results were analyzed using the following:  a. Unpaired t test; b. Pearson chi-square test; c. Mann–Whitney U test | | | | | |

| **Table S2.** Associations between the Paper Tug Test (Kami-Tsumami test) and muscle weakness, physical performance, muscle mass, sarcopenia, and frailty | | | | | |
| --- | --- | --- | --- | --- | --- |
| **Outcome** | n (%) | Crude OR | | Adjust OR^†^ | |
|  |  |  | *P* |  | *P* |
| **Low muscle strength** | 106 (30.8%) | **-** | ***-*** | - | ***-*** |
| Able to tug | 45 (39.1%) | 1.00 (reference) | ***-*** | 1.00 (reference) | ***-*** |
| Unable to tug | 61 (26.6%) | 0.57 (0.35-0.91) | ***0.019*** | 0.66 (0.39-1.14) | ***0.136*** |
| **Low physical performance** | 24 (7.0%) | **-** | ***-*** | - | ***-*** |
| Able to tug | 12 (10.4%) | 1.00 (reference) | ***-*** | 1.00 (reference) | ***-*** |
| Unable to tug | 12 (5.2%) | 0.48 (0.21-1.09) | ***0.080*** | 0.70 (0.26-1.83) | ***0.463*** |
| **Low muscle mass** | 143 (41.6%) | **-** | ***-*** | - | ***-*** |
| Able to tug | 59 (51.3%) | 1.00 (reference) | ***-*** | 1.00 (reference) | ***-*** |
| Unable to tug | 84 (36.7%) | 0.55 (0.35-0.87) | ***0.010*** | 0.59 (0.36-0.99) | ***0.045*** |
| **Sarcopenia** | 70 (20.3%) | **-** | ***-*** | - | ***-*** |
| Able to tug | 27 (23.5%) | 1.00 (reference) | ***-*** | 1.00 (reference) | ***-*** |
| Unable to tug | 43 (18.8%) | 0.75 (0.43-1.30) | ***0.308*** | 0.97 (0.52-1.80) | ***0.929*** |
| Abbreviations*:* OR, odds ratio; CI, confidence interval; ASM, appendicular skeletal muscle mass  Bold typeface indicates statistical significance *(P<0.05)*  ^†^Ratios and 95%CIs were adjusted by propensity score calculated from potentially confounding factors as follows: age, sex, body mass index, years of education, living alone, mini-mental state examination score, and chronic diseases (hypertension, diabetes mellitus, osteoporosis, dyslipidemia, malignant neoplasm, heart disease, stroke, and chronic renal failure). | | | | | |
